# Pathological investigations and correlation research of microfibrillar-associated protein 4 and tropoelastin in oral submucous fibrosis

**DOI:** 10.1186/s12903-021-01962-w

**Published:** 2021-11-19

**Authors:** Binjie Liu, Wenqun Gou, Hui Feng

**Affiliations:** 1 Department of Oral Medicine, Xiangya Stomalogical Hospital, Central South University, Changsha, China; 2 Changsha Stomatological Hospital, Changsha, China

**Keywords:** Microfibrillar-associated protein 4, Tropoelastin, Oral submucous fibrosis, Immunohistochemistry, Pathological characteristic

## Abstract

**Background:**

Oral submucous fibrosis (OSF), distinguished by abnormal collagen deposition, is a potentially malignant disorder with 4.2% (95% CI 2.7–5.6%) of malignant transformation and rising global prevalence. However, the precise pathogenesis and effective treatment remain elusive and controversial despite the abundance of literature on this topic. Therefore, it is crucial to explore the clinicopathological characteristics and potential markers for the diagnosis and prognosis of OSF. The objective of this study was to evaluate the influence and correlation of Microfibrillar-associated protein 4 (MFAP4) and tropoelastin (TE) in the development of OSF patients.

**Material and methods:**

Clinicopathological factors, hematoxylin–eosin (HE) and Masson trichome staining, immunohistochemical characteristics and the correlation between MFAP4 and TE were recorded and compared among different stages of OSF progression among cases (n = 60) and controls (n = 10). Student's t test, ANOVA analysis, and the chi-square test were performed to compare the categorical variables for clinicopathological characteristics and the expression level of MFAP4 and TE between the fibrotic and normal tissues. Correlation analysis of MFAP4 and TE was performed using Pearson's correlation test and linear regression.

**Results:**

MFAP4 and TE proteins are upregulated and increased gradually in patients with varying stages of OSF, relative to the control group. Furthermore, statistical analyses revealed that the expression level of MFAP4 was positively associated with TE, with a Pearson correlation coefficient of 0.3781 (*p* = 0.0048). Clinically, we found that OSF affected more males than females, with a ratio of 29:1. The age range was 16–60 years, and the mean age was 36.25 ± 10.25 years. In patients younger than 40 years, the positive expression rate of MFAP4 and TE was higher than in those over 40 years. All OSF cases had chewed areca nut, with 51.67% smoking tobacco.

**Conclusions:**

Our study elucidates that the accumulation of MFAP4 and TE proteins may play a vital role in the occurrence and development of OSF and may be promising candidate moleculars for prevention, diagnosis, and treatment strategies for OSF in the future.

## Introduction

Oral submucous fibrosis (OSF) is a chronic, irreversible, persistent progressive, and potentially malignant disease with a malignant transformation rate of 4.2% (95% CI 2.7–5.6%) [1–3]. Currently, the significant accumulation of the extracellular matrix (ECM) in both OSF and other fibrotic diseases has attracted considerable attention. The defective ECM dynamics stimulated by arecoline leads to a decline in collagen clearance and an increase in collagen synthesis during the development of fibrotic diseases [4]. Inspired by this, by proteomic analysis, our previous preliminary findings showed that MFAP4, an extracellular matrix protein, was notably upregulated in OSF tissues [5]. MFAP4 is a ubiquitous protein that plays an increasingly noteworthy part in elastin fiber formation and ECM remodeling processes during vascular injury and multitudinous fibrotic diseases, including myocardium, liver, joint and renal fibrosis [6–12]. In viral hepatitis and cirrhosis patients, transcription and protein levels experiment and histochemical analysis showed that MFAP4 levels increased significantly while progressing from non-fibrosis to severe stages [13, 14]. Moreover, extensive research has shown that serum MFAP4 levels can be used as a diagnostic predictor of varying degrees of liver cirrhosis and liver fibrosis [15]. Additionally, the findings revealed that plasma and atrial MFAP4 protein expression was elevated in rats with atrial fibrosis [16]. Although MFAP4 is a crucial ECM protein in the development of various fibrotic diseases, the expression and distribution of MFAP4 in various stages of OSF has not yet been fully elucidated.

MFAP4 protein is capable of binding specifically to TE proteins and actively promotes TE self-assembly [17]. TE, also an extracellular matrix protein, is a soluble precursor of elastin and the principal structural constituent of microfibrils in elastic fibers. TE protein is excreted by fibroblasts and vascular smooth muscle cells (VSMCs) [18, 19]. Moreover, upregulated TE protein is involved in the process of fibrosis by influencing fibroblasts, inflammatory cells, and angiogenesis in skin, lung, and liver fibrosis [14, 20–23]. However, the expression of TE protein and the correlation between MFAP4 and TE in the development of OSF remains unknown.

Therefore, in this study, appropriate samples with definite diagnosis were selected by H&E staining and Masson staining; and changes in MFAP4 and TE expression in OSF patients were detected by immunohistochemistry. Finally, the clinical information of the 60 patients was statistically analyzed. The purpose of this study was to visualize the presence and correlation of MFAP4 and TE and their role in the progression of OSF, and to identify these two proteins as promising candidates for the diagnosis and therapy of OSF patients in the future.

## Materials and methods

### Sample selection

All clinical samples and data were collected from patients at the Department of Maxillofacial Surgery, Oral Medicine and Pathology, Xiangya Stomatological Hospital and Xiangya Hospital, Central South University, Hunan, China. The samples were collected between 2014 and 2020. Informed consent was signed by all study participants, and the research was authorized by the Institutional Research Ethics Committee of Xiangya Stomatological Medical College (approval number 20200034). No patients had any medical history within the past 2 months, a history of disease treatment or any systemic diseases. Basic patient information, past history, and bad habits were recorded. A total of 10 young Chinese individuals with no history of betel quid chewing and smoking without current or prior oral mucosal diseases provided the control group. The study was carried out with 60 patients diagnosed with OSF having relevant clinical manifestation and pathological characteristics. These OSF cases were graded according to Wollina, U and Verma, S. B. staging [24]. In the early stage, the patients had no obvious discomfort or mild pain during eating. Histological analysis of the epithelial layer showed no significant change compared to that in normal tissues. Slight hyalinization, mildly dilated and congested blood vessels and inflammatory cell infiltration were found in the juxta-epithelial area. In the mid-stage of OSF, patients experience obvious irritation and pain while eating and, demonstrated restricted mouth opening, within a range of two fingers. Localized hyaline degeneration, increased collagen accumulation, and significantly smaller and decreased blood vessels were observed in the juxta-epithelial area. In the late stage of fibrosis, patients develop trismus and even difficulty in eating, speech, chewing, and swallowing. Obvious epithelial atrophy, vacuolar degeneration, large amounts of collagen accumulation, diffuse hyaline degeneration and lots of narrowed or occluded blood vessels were displayed. The diagnoses were assessed and independently verified under 4×, 10×, and 40× magnifications by two experienced pathologists (Zhigang Yao and Long Li) of Xiangya Stomatological Hospital.

### Hematoxylin–eosin and Masson trichome staining

H&E staining was conducted according to the standard procedure and was applied to observe the morphological changes in control and OSF oral mucosa tissue. Masson staining via a classic three-color method was used to examine and define the pathological phase of the OSF specimen and observe the distribution of collagen under the microscope. The slides were then stored at room temperature. The OSF sections were graded by two observers to overcome inter-observer variation. The Masson's trichrome-stained slide for OSF was graded according to the following three stages: (1) early stage, inflammatory cell infiltration was observed; the connective tissue of the lesion showed some tiny collagen fibers and fibrosis limited to the lamina propria; (2) mid-stage, the hyaline changes of collagen fibers were aggravated, showing bands or strips, and expanded to the superficial region of the muscle bundle; the vascular lumen was significantly reduced; and (3) late stage, cortical atrophy; fibrosis is observed in the deeper regions of the muscle bundle; all collagen fibers become transparent; normal structures disappear completely; and blood vessels are constricted or occluded [25]. The collagen was stained blue while the muscle took a brilliant red color.

### Immunohistochemistry

The tissue samples were collected and fixed with paraformaldehyde, embedded in paraffin, and sectioned for immunohistochemical reaction (IHC). After deparaffinization and rehydration, the tissue sections were subjected to antigen retrieval and blocked with 3% hydrogen peroxide to interdict endogenous peroxidases. Primary antibodies were diluted with 3% BSA solution at a ratio of 1:500 for both MFAP4 (ab103925; Abcam) and TE (ab21600; Abcam). The histological sections were incubated with the labeled primary antibodies overnight at 4 °C. Subsequently, the appropriate secondary antibodies were incubated for 30 min followed by three PBS buffer washes for incubation with a fresh solution of 2,3-diaminobenzidine (DAB) hydrochloride substrate.

The stained slides were allocated to observers in a double-blind form to eliminate inter-observer bias and assessed under a light microscope. To calculate the positive rate of each tissue, five random areas within a section were selected at 100× magnification, and the staining intensity and the range of staining were estimated. The immuno-staining score was measured by the following two rules: (1) staining intensity corresponding to score 1 (0, negative; 1, weak; 2, moderate; 3, strong). (2) the range of staining corresponding to score 2 (0, < 5%; 1, 6–25%; 2, 26–50%; 3, 51–75%; 4, > 75%). The total score of the staining intensity was calculated by multiplying score 1 by score 2 and was subjected to statistical analysis [2].

### Statistical analysis

Data were evaluated using the GraphPad Prism 8. Student's t test and ANOVA analysis were used to assess the expression of MFAP4 and TE proteins in all tissue samples. Chi-square test for categorical variables was conducted for clinicopathological characteristics of the OSF samples and the differential expression of MFAP4 and TE between the OSF and control tissues. The correlations between MFAP4 and TE were assessed using Pearson's correlation test and linear regression. For all experiments, significance was evaluated as *p < 0.05; **p < 0.01; ***p < 0.001; and ns > 0.05.

## Results

### Clinical specimen classification and pathological feature evaluation

**Fig. 1:**
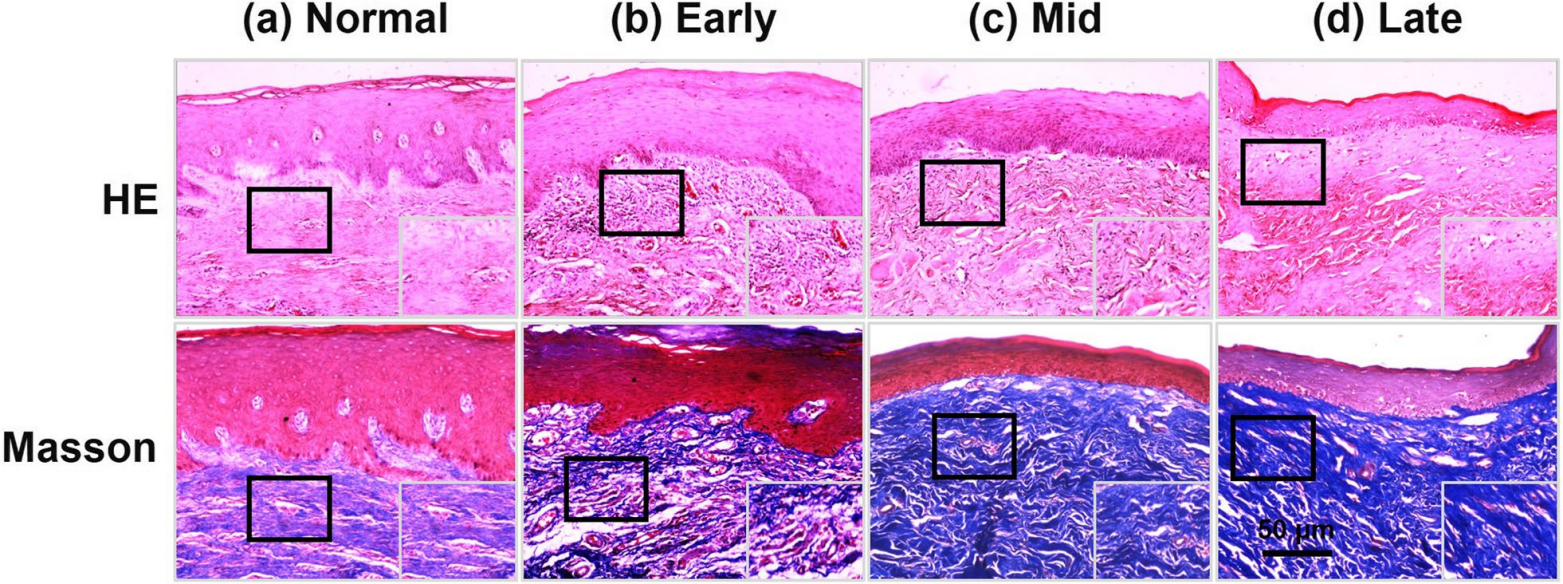
H&E and Masson staining. **a** The structure of normal oral mucosa tissue. **b** Mild inflammatory infiltrates of juxta-epithelial area and blood vessels in the early phase of OSF. **c** Limited hyaline degeneration, obvious collagen accumulation with stripy or banded fibers, and significantly reduced lumen size in the mid stage of OSF. **d** Visibly atrophied epithelium, epithelial vacuolar degeneration, plate-like collagen fibers, and diffused hyaline degeneration leading to narrowed or occluded vascula in the late stage of OSF

Ten control and 60 OSF mucosal tissue samples were collected. OSF tissues (13 early, 26 middle, and 21 late OSF tissues) were classified by H&E and Masson staining. In the early phase (Fig. 1a, b), the epithelial layer showed no significant change compared that in control tissues. However, some fine collagen fibers maintaining their original structure were present and were properly looser in the juxta-epithelial area. In the meantime, the majority of the blood vessels were normal, but some were dilated and congested, and some lesions displayed features of slight inflammation in the sub-epithelial tissue. In the mid-stage of OSF, the lesion area displayed limited hyaline degeneration, while collagen fibers in the lamina propria were strip-like or banded, and then lumen diameter of the vessels decreased was clearly decreased (Fig. 1c). In the late stage of fibrosis, epithelial atrophy could be seen in the epithelial layer, and vacuolar degeneration occured in each layer of the epithelium, especially in the spinal cell layer. All fibrous collagen were glass-like, the normal structure had disappeared completely, and the blood vessels were narrowed or occluded (Fig. 1d).

### Increased expression of MFAP4 and TE proteins is involved in the progression of OSF

**Fig. 2:**
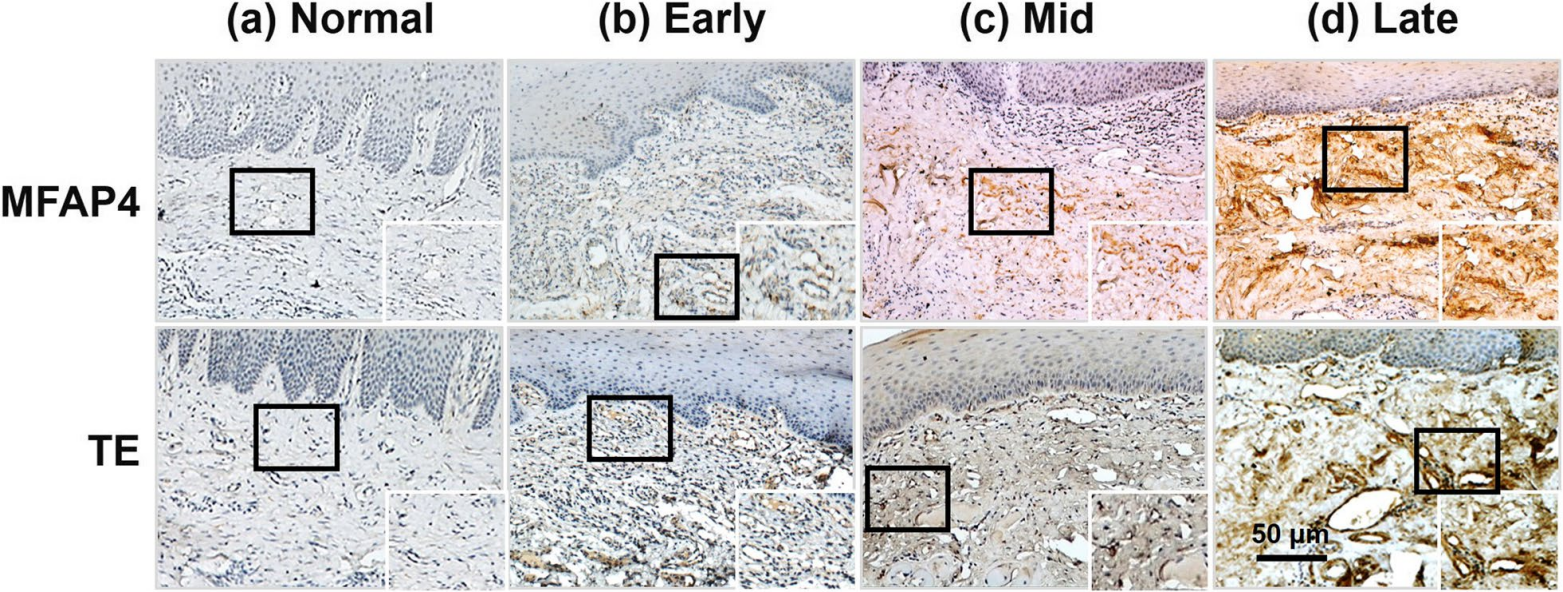
Immunohistochemistry (IHC) analysis of MFAP4 and TE in control and oral submucous fibrosis (OSF) oral mucosa tissues. IHC images showing the incremental expression of MFAP4 and TE in the control l (**a**) and early (**b**), middle (**c**) and late stages (**d**) of OSF

**Fig. 3:**
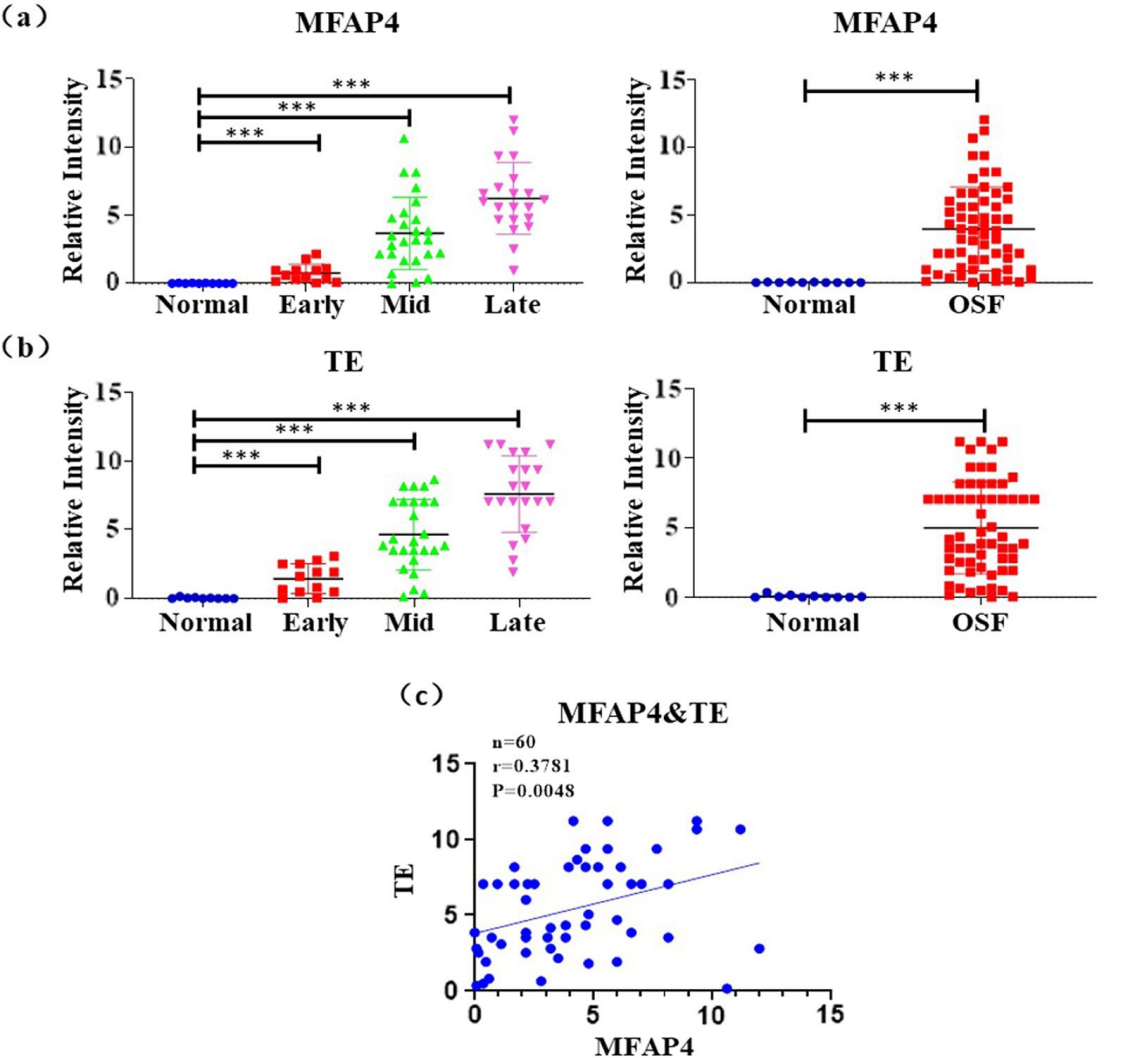
Statistical results of the IHC intensity scores (**a**, **b**) and correlation analysis (**c**) displaying the positively correlation of MFAP4 and TE in normal oral mucosa and different stages of OSF tissues. *p < 0.05, **p < 0.01, ***p < 0. 001

To investigate the relationship between OSF progression and MFAP4 expression in the ECM, clinical OSF samples at different stages were analyzed by immunohistochemistry. We observed the collagen deposition in various pathological stages of OSF using Masson staining. The findings indicated more intense blue staining in the submucosa due to collagen deposition in all OSF tissues compared with that in control tissues. As the degree of fibrosis increased, the intensity of blue staining in the connective tissue increased (Fig. 1).Subsequently, we discovered that strong MFAP4 immunoreactivity was identified in the subepithelial connective tissues of these OSF tissues at different stages (n = 70, *p* = 0.0007) (Figs. 2 and 3a). Moreover, the expression level of MFAP4 protein was slightly increased in the early stage (n = 13, *p* = 0.0015) and mid-stage (n = 26, *p* = 0.0001) and significantly increased in the late stage (n = 21, *p* < 0.0001).

Since TE and MFAP4 have been reported to participate jointly in elastin assembly and are closely related to the progression of many fibrotic diseases [14, 17, 21, 26]. We studied the expression and distribution of TE proteins in the normal and fibrotic tissues via immunohistochemical staining methods. To our knowledge, the expression of TE proteins at different OSF stages has not been reported. Our analysis demonstrated increase, compared to control tissue, in the intensity of TE in submucosa area in the early stage (n = 13, *p* = 0.0010), mid-stage (n = 26, *p* < 0.0001) and late-stage tissues (n = 21, *p* < 0.0001), which is consistent with the expression pattern of MFAP4, as illustrated in Figs. 2 and 3b (n = 70, *p* < 0.0001). MFAP4 and TE proteins were also occasionally expressed in blood vessels.

To investigate the potential correlations betweenMFAP4 and TE protein expression in OSF tissues, the Pearson correlation test was used to establish the correlation model. The statistical analyses showed that the expression level of MFAP4 was positively associated with TE, with a Pearson correlation coefficient of 0.3781 (*p* = 0.0048) (Fig. 3c). We demonstrated a significant positive correlation between MFAP4 and TE expression in the OSF submucosa area, suggesting that they are closely associated with the progression of OSF.

### Clinicopathological features of OSF

To investigate the clinicopathological features, clinical information of 60 patients with OSF was obtained. We found that OSF affects more males than females, with a ratio of 29:1. The age range was 16–60 years, and the mean age was 36.25 ± 10.25 years. OSF was most frequent in the age range of 31–40 years (36.67%). Consistently, the positive expression rate of MFAP4 and TE in patients aged less than 40 years was higher than that in those over 40 years. Of the OSF cases, all had chewed betelnut, with 51.67% smoking tobacco (Table 1).

**Table 1:** The evaluation of clinicopathological features associated with the expression of MFAP4 and TE of OSF patients

Characteristics	No of sample (%)	MFAP4		*p* value	TE		*P* value
		Negative	Positive		Negative	Positive	
*Sex (patients)*							
Male	58 (96.7)	15	43	ns	8	50	ns
Female	2 (0.03)	0	2		1	1	
*Age (patients)*							
< 40	42 (70)	7	35	0.0475*	3	39	0.0164*
≥ 40	18 (30)	8	10		6	12	
*Stage*							
Normal	10 (14.3)	10	0	< 0.0001***	10	0	< 0.0001***
Early	13 (18.6)	10	3		6	7	
Mid	26 (37.1)	4	22		3	23	
Late	21 (30)	1	20		0	21	
*Betel quid chewing*							
Yes	60 (100)	19	41	ns	15	45	ns
No	0 (0)	0	0		0		
*Smoking*							
Yes	31 (51.7)	10	21	0.2377	6	25	0.4743
No	29 (48.3)	5	24		3	26	
*Clinical feature*							
Restriction of mouth opening	35 (58.3)	8	27	0.6227	4	31	0.4367
Burning senseation	10 (16.7)	2	8		2	8	
Blister or ulcer	14 (23.3)	2	12		3	11	
Eating stimulation pain	21 (35)	2	19		1	20	

## Discussions

Recent reports have shown that the oral mucosa has clinically and pathologically progressed from normal to OSF in some betel nut chewing enthusiasts [27, 28]. However, the molecular mechanism of the initiation and progression of OSF from normal tissue to the fibrotic state remains unclear. Earlier studies have demonstrated that the basic pathological process of OSF is microtrauma caused by the continuous friction of betel nut crude fibers, leading to the infiltration of inflammatory cells in the proximal epithelial area, and the secretion of cytokines. Subsequently, these cytokines act on fibroblasts, promote fibroblast proliferation, and stimulate the production of collagen fibers and ECM in large quantities. To the best of our knowledge, defective ECM dynamics is a critical factor during OSF progression. These classic theories implicate an underlying connection between the pathological processes of OSF and ECM.

MFAP4 is a key ECM. Current research has shown that MFAP4 expression is correlated with a number of functions, including coagulation, angiogenesis, tissue growth and remodeling and innate immunity [29–31]. Furthermore, increased MFAP4 protein levels play an extremely important role in the development of fibrotic diseases, including heart, liver, joint and kidney fibrosis [10, 13, 14, 16, 25, 32, 33]. However, whether MFAP4 is related to the OSF procedure has not been intensively investigated. Previously, though proteomic analysis, we reported research reported that MFAP4 was notably upregulated in OSF tissues [5]. In the present study, we found that the MFAP4 protein was overexpressed in most OSF samples compared to control samples. More importantly, the increase was much more dramatic in the late stage of OSF than in the early stage. Therefore, these results suggested that MFAP4 plays an important role in the pathogenesis of OSF and has the potential to be used as a diagnostic and therapeutic marker of OSF.

In addition, TE, a soluble precursor of elastin and another key ECM protein, is believed to be a pro-fibrosis factor. Previous studies have indicated that the expression of TE was significantly increased in asbestos-induced airway fibrosis in rats and in fibrotic tissues such as skin white lines, fine-line scars, hypertrophic scars, and keloids, and is closely correlated with pulmonary fibrosis and skin fibrosis [20–22]. Moreover, MFAP4, together with TE, participates in the assembly of elastin and the two proteins are upregulated in liver fibrosis and cirrhosis, and can serve as a biomarker of liver fibrosis [14, 17, 23]. In liver fibrosis, TE acts directly on fibroblasts and inflammatory cells, and promotes angiogenesis, thus promoting the development of fibrosis [34]. In our study, we observed that the expression of TE was significantly higher in the OSF samples than in the control samples, in line with other fibrotic diseases. Further, we demonstrated that the expression of TE was clearly enhanced in the later stage of OSF compared to the early stage. We then analyzed the relationship between MFAP4 and TE by Pearson's correlation test and linear regression and concluded that the expression level of TE was positively associated with MFAP4 protein, indicating that MFAP4 and TE may regulate the development of OSF together.

Finally, we assessed the connection between the expression of MFAP4 and TE and the clinical data of OSF individuals. There was a negative association between MFAP4 and TE expression levels and sex, smoking and various clinical features. Statistically significant correlations were found between the expression of the two proteins and chewed betel nut, age, and the severity of OSF (Table 1). The disease tends to occur in young and middle-aged individuals. Consistently, the positive expression rate of MFAP4 and TE in patients less than 40 years of age was higher than that in those over 40 years.

Meanwhile, our study needs to be further deepened and enhanced in the future. Our current study was the small number of included subjects and the sample size limited the correction of confounding factors including age, gender, oral health status, smoking and alcohol consumption. In the future, more investigation is needed to determine the role and detailed mechanism of MFAP4 and TE in the development of OSF.

In conclusion, we report that increased expression of MFAP4 and TE are associated with OSF. Furthermore, the expression of MFAP4 and TE proteins increased gradually in early, middle and late stages OSF, and there was a strong correlation between the expression of MFAP4 and TE in OSF tissues. Therefore, we conclude that the MFAP4 and TE proteins may be closely related to the development of OSF and have potential clinical value as novel biomarkers for the diagnosis and therapy of OSF.
